# DIETERICH’s disease of both the third and the fourth metacarpal head in a teenage boy: case report and review of literature

**DOI:** 10.1080/23320885.2024.2316026

**Published:** 2024-02-15

**Authors:** Mehdi Drissi Kaitouni, Aleid C. J. Ruijs

**Affiliations:** a Department of Orthopaedic Surgery, Centre Hospitalier Wallonie Picardie, Tournai, Belgium; b Department of Orthopaedic Surgery, Université Catholique de Louvain St Luc, Brussels, Belgium

**Keywords:** Dieterich’s disease, adolescent, metacarpal bone, avascular necrosis

## Abstract

Dieterich’s disease or avascular osteonecrosis of the metacarpal head is rare and not often described in the literature. It affects typically the middle of metacarpal bones and can occur at all age groups. A case of a teenager with chronic pain of the third and fourth metacarpal head is presented.

## Introduction

Dieterich’s disease or avascular osteonecrosis of the metacarpal head is a rare disease. It occurs in both adults and children, with more than 50% of reported cases in the literature aged under 20 years old [1]. Usually chronic pain, stiffness, and limitation in activities of daily life or sports activities lead the patient to consult at the outpatient clinic. Although conservative treatment is currently the gold standard, several surgical treatments have been described. Multiple locations in the same patient of avascular osteonecrosis are rare.

## Case report

**Figure 1.**
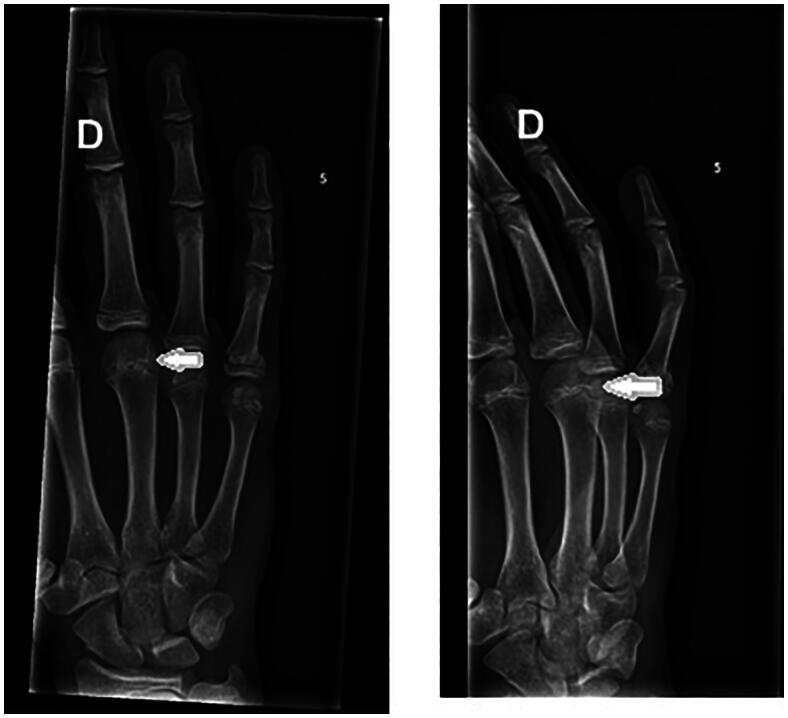
Radiograph July 2022, osteolysis of the third metacarpal head with sclerosis.

**Figure 2.**
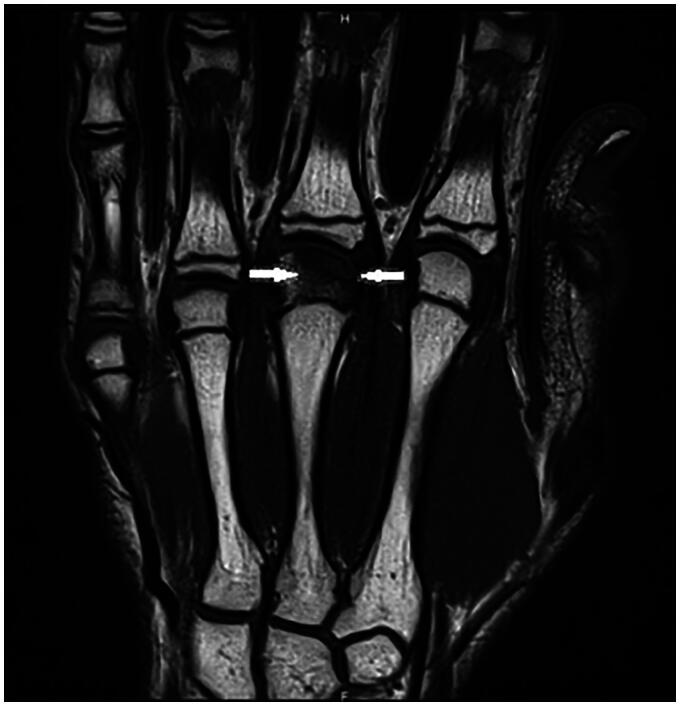
First MRI, July 2022. Subchondral fracture of the distal epiphysis of the third metacarpal head, with probable avascular necrosis and preserved viability in T1 weighted images.

**Figure 3.**
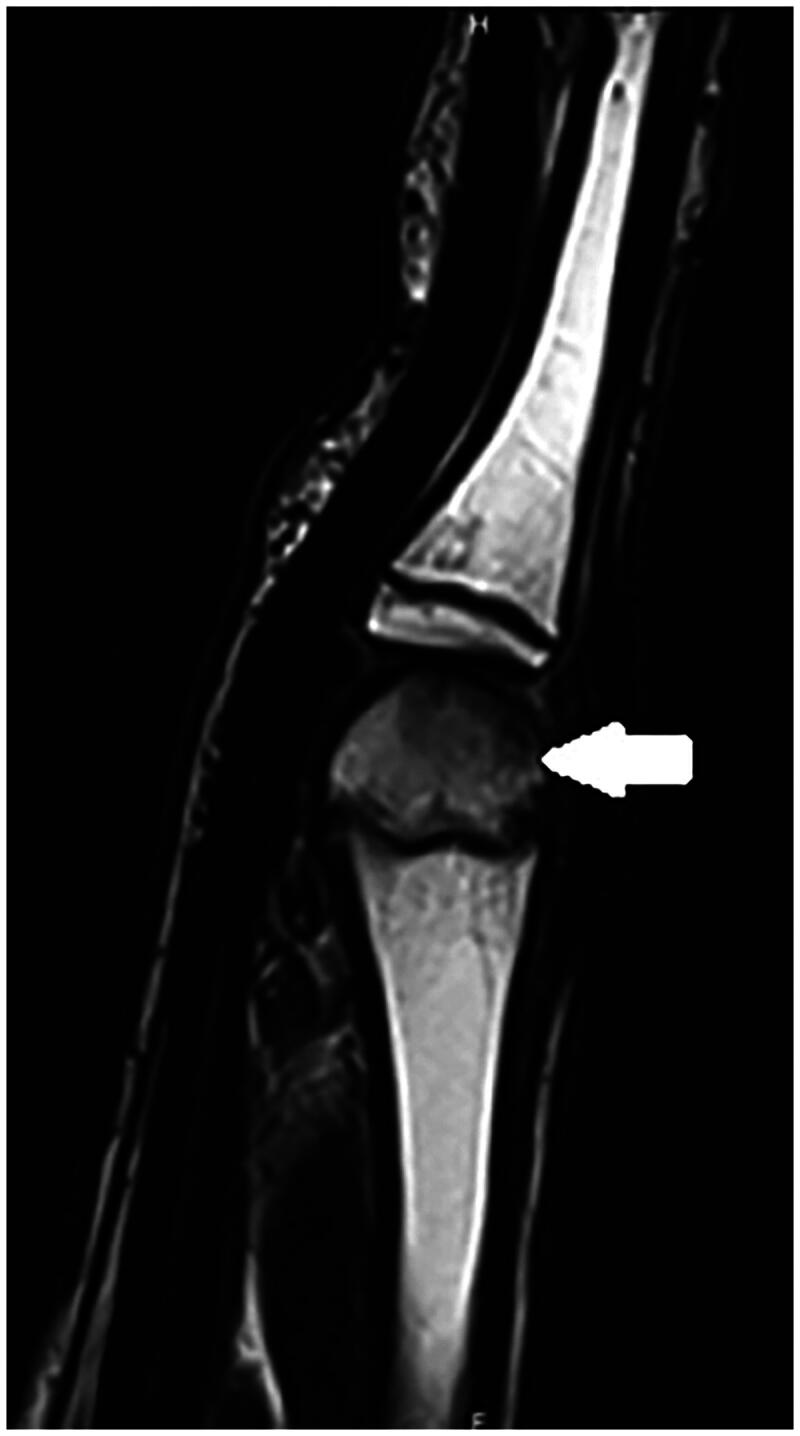
First MRI, July 2022. Subchondral fracture of the distal epiphysis of the third metacarpal head, with probable avascular necrosis and preserved viability in T1 weighted images.

**Figure 4.**
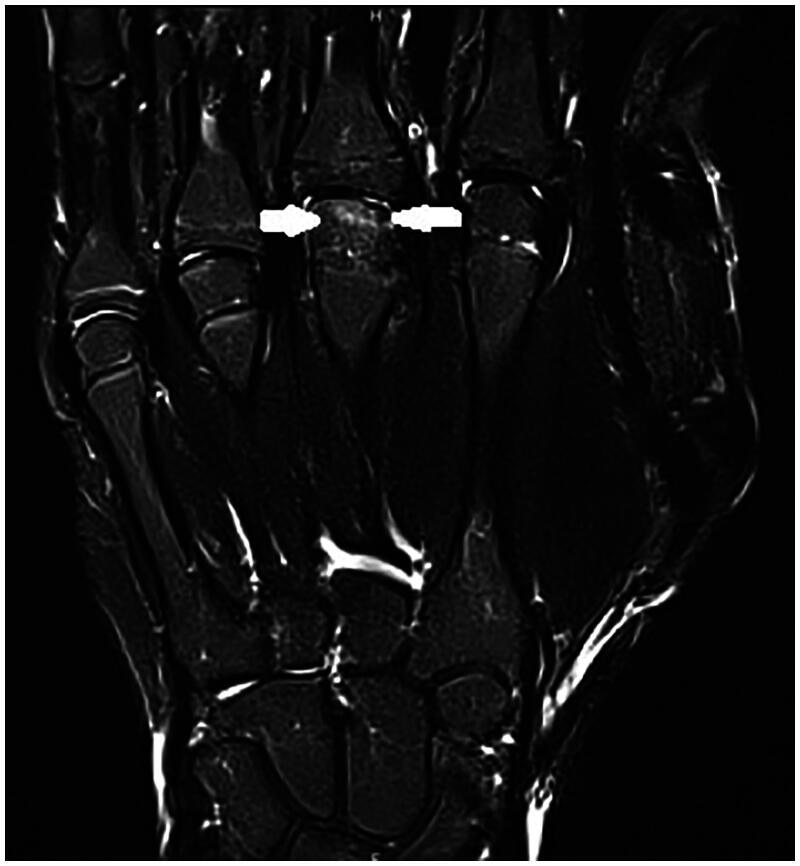
Second MRI, October 2022, vascularization seems preserved in T2 weighted images.

**Figure 5.**
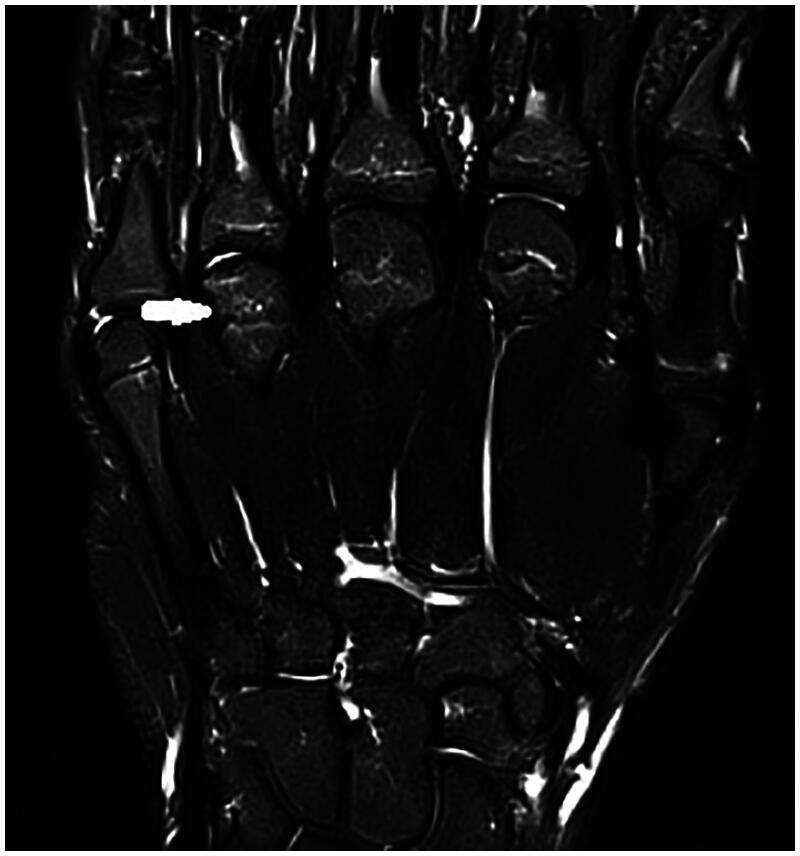
Third MRI January 2023. Apparition of signal abnormality of the fourth metacarpal head in the MRI T1 weighted images.

**Figure 6.**
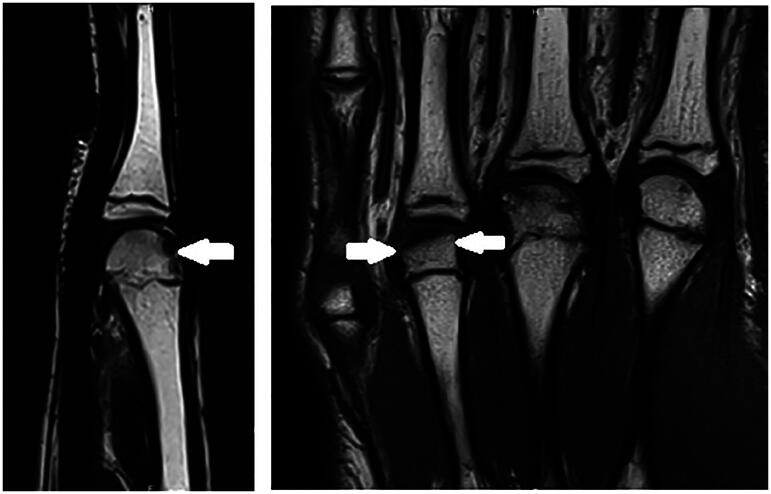
Third MRI January 2023. Apparition of signal abnormality of the fourth metacarpal head in the MRI T1 weighted images.

**Figure 7.**
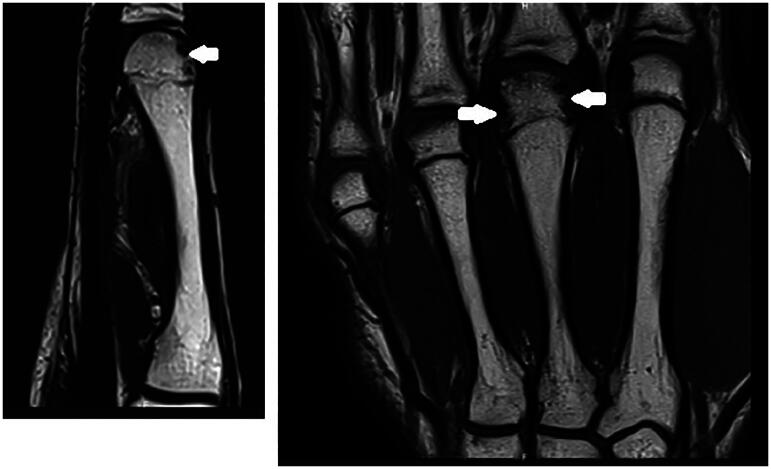
MRI April 2023. Normalization of the abnormalities, in T1 weighted images.

A 14-year-old boy, right-handed and frequent (three to four times per week) basketball player presented with spontaneous pain in the third metacarpophalangeal (MCP) joint region of his right hand. This pain had been present for over 3 months; start of symptoms was March 2022. Previously, he had been treated conservatively for a fracture of the first phalanx of the right little finger (January 2022). On examination of his right hand, we note no abnormalities and no swelling, the palpation of the third metacarpal head was painful. The radiograph revealed osteolysis of the third metacarpal head with sclerosis (Figure 1). An MRI study confirms subchondral fracture of the distal epiphysis of the third metacarpal head, with probable avascular necrosis and preserved viability (Figures 2 and 3). The initial treatment consisted of day and night immobilization in a thermoplastic splint and analgesics if needed. Three months later, there is clinical improvement and absence of pain. The MRI study noted a decrease in oedema of the distal epiphysis, vascularisation seems preserved and there is a slight loss of head sphericity compared to previous examination (Figure 4). At 6 months, examination is still good, and shows a maximal range of motion, no pain, no swelling, no strength, or sensibility deficit. A repeated MRI study, however, confirms the persistence of signal abnormalities of the third metacarpal head in the right hand, AND the apparition of signal abnormality of the fourth metacarpal head (Figures 5 and 6). An additional treatment of aspirin 80 mg once daily was proposed but refused by the parents. It was advised to continue the conservative treatment with immobilisation. At 9 months, the clinical findings remained similar, no pain and a maximal range of motion. The young boy returned to play basketball. Radiological evolution is characterized by a small stable flattening of the radial side of the third metacarpal head in the right hand. We note a marked reduction in the linear signal abnormalities of the subchondral bone. Also, there is a regression of the subchondral abnormalities of the fourth metacarpal head (Figure 7).

## Discussion

Dieterich’s disease or avascular necrosis of the metacarpal head appears mostly in younger population [1]. The main symptoms described in literature are swelling, pain, limitation of range of motion. It can occur secondary to trauma, systemic disease like systemic lupus erythematosus, steroid use, congenital short digits, or an atypical anatomical epiphyseal blood supply. The lack of vascularisation to the metacarpal head may be due to the absence of large nutrient vessels in 35% of specimens, as mentioned by Wright and Dell [2]. Mostly it occurs spontaneously [3–5] and may lead to progressive destruction of the metacarpophalangeal joint and hand function [6]. In the initial assessment, radiographs and MRI studies are performed to confirm the avascular necrosis. MRI examination is the most sensitive exam and shows an area of low signal intensity in T1 and in T2 sequences displaying a distinctive pattern designated the double-line sign. Radiographs generally reveal osteolysis of the metacarpal head with sclerosis or cystic lesions [7–10]. In our case, the basketball practice and the history of finger fracture may explain the apparition of the disease. In fact, basketball is a sport which can create micro traumatisms in the fingers and create a deficit in the periosteal vasculature. However, imaging and clinical symptoms are not necessarily correlated.

Fan explains in his review of 45 studies with 55 patients, that an early diagnosis will provide an optimal clinical outcome, restoring joint activity and resolving pain. The review shows that of eighteen cases treated conservatively, ten healed successfully [6].

Literature confirms that the most common site of avascular necrosis is the third metacarpal head. Other articles, describe the lesion in other metacarpal heads as well [4]. The treatment can be often conservative with painkillers and rest, or more rarely surgical. The duration of the conservative treatment and its management is not clear yet and depends on surgeon experience and the clinical and radiological evolution. The duration of nonoperative treatment should be longer for adolescents, especially for those with multiple lesions, as they explain in their review of literature [6]. In our case, the adolescent boy healed completely and had a complete range of motion without pain after 9 months. Most cases of successful nonoperative treatment were juveniles, which might be related to the regenerative potential of the growing bone.

In case of a surgical treatment, the status of the cartilage and extend of its damage, determines the type of surgery chosen. Surgical procedures described in literature are for example: curettage and bone grafting [11]; denervation of the metacarpal joint [8]; osteochondral auto graft transfer system [12]; pyrolytic carbon hemiarthroplasty of the metacarpal head [13]; osteochondral mosaicplasty [14]; and joint fusion [6]. All these procedures are case reports; there is no consensus and there are no long-term results available in the literature concerning surgical treatment of the Dieterich’s disease.

## Conclusion

In conclusion, Dieterich’s disease or avascular necrosis of the metacarpal head, is a rare condition, the most common region affected is the head of the third metacarpal and often due to a trauma. The better treatment in terms of outcomes is still unclear conservative or surgical, but we believe that we have to stick to the clinical characteristics of the patient, even more with children or teenagers, who have the advantage of the regenerative potential of growing bones.

## Informed consent

The patient and his parents provided written consent for the publication of the material. He was informed that he would not be identified and that his data would be completely anonymized for publication.
